# Genetic-interaction screens uncover novel biological roles and regulators of transcription factors in fission yeast

**DOI:** 10.1093/g3journal/jkac194

**Published:** 2022-08-04

**Authors:** Kate Chatfield-Reed, Kurtis Marno Jones, Farah Shah, Gordon Chua

**Affiliations:** Department of Biological Sciences, University of Calgary, Calgary, Alberta T2N 1N4, Canada; Department of Biological Sciences, University of Calgary, Calgary, Alberta T2N 1N4, Canada; Department of Biological Sciences, University of Calgary, Calgary, Alberta T2N 1N4, Canada

**Keywords:** *Schizosaccharomyces pombe*, transcription factor, synthetic lethality, synthetic dosage lethality

## Abstract

In *Schizosaccharomyces pombe*, systematic analyses of single transcription factor deletion or overexpression strains have made substantial advances in determining the biological roles and target genes of transcription factors, yet these characteristics are still relatively unknown for over a quarter of them. Moreover, the comprehensive list of proteins that regulate transcription factors remains incomplete. To further characterize *Schizosaccharomyces pombe* transcription factors, we performed synthetic sick/lethality and synthetic dosage lethality screens by synthetic genetic array. Examination of 2,672 transcription factor double deletion strains revealed a sick/lethality interaction frequency of 1.72%. Phenotypic analysis of these sick/lethality strains revealed potential cell cycle roles for several poorly characterized transcription factors, including SPBC56F2.05, SPCC320.03, and SPAC3C7.04. In addition, we examined synthetic dosage lethality interactions between 14 transcription factors and a miniarray of 279 deletion strains, observing a synthetic dosage lethality frequency of 4.99%, which consisted of known and novel transcription factor regulators. The miniarray contained deletions of genes that encode primarily posttranslational-modifying enzymes to identify putative upstream regulators of the transcription factor query strains. We discovered that ubiquitin ligase Ubr1 and its E2/E3-interacting protein, Mub1, degrade the glucose-responsive transcriptional repressor Scr1. Loss of *ubr1^+^* or *mub1^+^* increased Scr1 protein expression, which resulted in enhanced repression of flocculation through Scr1. The synthetic dosage lethality screen also captured interactions between Scr1 and 2 of its known repressors, Sds23 and Amk2, each affecting flocculation through Scr1 by influencing its nuclear localization. Our study demonstrates that sick/lethality and synthetic dosage lethality screens can be effective in uncovering novel functions and regulators of *Schizosaccharomyces pombe* transcription factors.

## Introduction

Transcription factors are an integral component of the response to external stimuli during growth and development (Spitz and Furlong 2012; Lambert *et al.* 2018). Signal transduction pathways impinge upon transcription factors often by posttranslational modifications to control their abundance, localization, and activity within the cell (Filtz *et al.* 2014; Weidemuller *et al.* 2021). The assembly and activity of sequence-specific transcription factors containing defined DNA-binding domains (referred to as transcription factors hereafter) at *cis*-regulatory sites give rise to combinatorial regulation of their target genes. The physical interactions between transcription factors, their regulators, as well as their target genes form a complex regulatory network that establishes the transcriptome of the cell.

The utilization of systematic genetics in characterizing transcription factors of several yeast species has made substantial contributions in determining their function and direct target genes (Chua 2013; Hughes and de Boer 2013; Rai *et al.* 2022). Mutant collections consisting of single deletion and overexpression transcription factor strains are a valuable resource to screen for specific phenotypes and carry out transcriptome profiling to determine differentially regulated genes (Chua *et al.* 2006; Homann *et al.* 2009; Yoshikawa *et al.* 2011; Vachon *et al.* 2013; Lohse *et al.* 2016). Despite the advances in deciphering transcriptional-regulatory networks from the analyses of these transcription factor mutant strains, 2 main limitations exist. First, the absence of a detectable mutant phenotype that could be used to assign a biological role remains elusive for certain transcription factors. For deletion strains, this could be due to transcription factors that are functionally redundant or not active under standard lab conditions (Chua *et al.* 2004; Vachon *et al.* 2013). Overexpression strains may not show a phenotype if ectopic expression of the transcription factor is not sufficient to induce its activity. Previous studies suggest that this may be the case as transcription factor overexpression strains that do not exhibit phenotypic activation tend to produce muted transcriptome profiles (Chua *et al.* 2006). Second, the identification of putative regulators of transcription factors is challenging when relying solely on the phenotypic analysis of single mutants.

Digenic interactions have the potential to identify novel biological roles and regulators of transcription factors not revealed in the phenotypic analyses of single transcription factor deletion or overexpression strains. A synthetic sick/lethal (SL) interaction is when the phenotype of a double deletion mutant has a greater growth defect than expected based on the growth of each single mutant (Costanzo *et al.* 2010). SL interactions indicate that the 2 genes buffer each other, either in the same pathway or through interactions between 2 pathways (Tong *et al.* 2001, 2004; Dixon *et al.* 2008; Costanzo *et al.* 2010; Ryan *et al.* 2012; Costanzo *et al.* 2016). Therefore, the biological roles of uncharacterized genes can be uncovered by their SL interactions with genes of known function through a guilt-by-association relationship. A synthetic dosage lethal (SDL) interaction is a growth defect observed from gene overexpression in a deletion background but not in wild type (Measday and Hieter 2002). SDL interactions usually involve 2 genes with opposing regulatory roles and result in the hyperactivation of a pathway that is detrimental to cell viability (Measday *et al.* 2005; Sopko *et al.* 2006). For example, SDL interactions occur if the deletion of a repressor further increases the activity of an overexpressed protein resulting in cell toxicity. SDL interactions can also involve genes whose products are components of the same complex. In this case, the SDL interactions are attributed to a disruption in the stoichiometry of the protein complex (Kaluarachchi Duffy *et al.* 2012).

In yeast, gene deletion and overexpression do not often result in a large fitness defect under standard laboratory conditions, and further perturbations are required to elucidate their role in the cell (Giaever *et al.* 2002; Sopko *et al.* 2006). SL and SDL synthetic genetic array (SGA) screens have been performed extensively in *S. cerevisiae* to further characterize gene function, facilitated by the availability of full-genome deletion and overexpression collections (Giaever *et al.* 2002; Sopko *et al.* 2006). Several large-scale SL screens, and smaller targeted screens focusing on specific cellular functions, such as transcriptional regulation or phosphorylation, have been performed in *S. cerevisiae* (Fiedler *et al.* 2009; Costanzo *et al.* 2010; Zheng *et al.* 2010; Costanzo *et al.* 2016). SDL screens have been used to systematically explore chromosome segregation, the transcriptome, the kinome, the ubiquitinome, and the acetylome in *S. cerevisiae* (Measday *et al.* 2005; Liu *et al.* 2009; Kaluarachchi Duffy *et al.* 2012; Sharifpoor *et al.* 2012; Youn *et al.* 2017).

In *S. pombe*, multiple SL SGA protocols have been developed (Roguev *et al.* 2007; Dixon *et al.* 2008; Rallis *et al.* 2017). These full-genome screens have generated partial genetic-interaction data for ∼50% of the genome (Roguev *et al.* 2007; Dixon *et al.* 2008; Ryan *et al.* 2012). However, these screens cover less than 15% of the possible pairwise interactions between transcription factors, leaving the genetic network of transcription factors relatively unexplored. In contrast, no protocol for SDL SGA screening currently exists in *S. pombe*. To date, 23 of 87 (26.4%) transcription factors have no assigned gene name and well-defined biological role, although the full complement of single deletion and overexpression strains has been phenotypically characterized under standard lab conditions (Kim *et al.* 2010; Wood *et al.* 2012; Hayles *et al.* 2013; Vachon *et al.* 2013). Furthermore, the identity of regulators is known for even fewer transcription factors than those that do not have an assigned gene name (Wood *et al.* 2012).

Here, we carried out SL and SDL SGA screens of *S. pombe* transcription factor genes to identify new biological roles and regulators, respectively. For the SL screens, we intercrossed 38 query and 91 array transcription factor deletion strains, which uncovered both novel and previously identified negative genetic interactions. These interactions resulted in several double mutants with additive effects of cell elongation involving transcription factors with both known and previously uncharacterized roles in the cell cycle. We also developed a modified SGA method to screen for SDL interactions in *S. pombe*, using transcription factor overexpression strains previously constructed in our lab (Kwon *et al.* 2012; Vachon *et al.* 2013). Fourteen transcription factor overexpression strains were crossed with a regulatory miniarray of 279 deletion strains consisting of genes encoding posttranslational modifying and signaling enzymes.

The SDL screens revealed known and putative novel regulators of the catabolite repression transcription factor Scr1, as well as the cell cycle transcription factors Yox1 and Tos4. During this study, we discovered that Scr1 has a novel role in the repression of flocculation. Four of the 6 confirmed SDL genes were shown to negatively regulate Scr1 either by protein degradation or by nuclear exclusion, and their gene deletion prevented flocculation under inducing conditions. Two novel putative regulators of Scr1 affecting degradation were the E3 ubiquitin ligase Ubr1 and the zf-MYND type zinc finger protein Mub1, both of which cause accumulation of the Scr1 protein when deleted. These results demonstrate the utility of SL and SDL SGA screens with transcription factor deletion and overexpression strains to further decipher the transcriptional-regulatory network of *S. pombe*.

## Materials and methods

### Strain construction and media

Supplementary Table 1 contains a list of yeast strains used in this study. Strains were grown on YES, EMM, or PMG medium supplemented with 225 mg/L each of adenine (A), leucine (L), uracil (U), histidine (H), and 15 µM thiamin where indicated. Matings were carried out on SPA medium supplemented with 45 mg/L each of A, L, U, and H. G418 (150 mg/L) and clonNAT (100 mg/L) were added to YES medium for the selection of strains containing ORFs replaced with the *KanMX4/KanMX6* and *NatMX4* cassettes, respectively. The YES low-glucose medium contained 0.08% glucose, and cells were induced to flocculate in flocculation-inducing medium (FIM) composed of 1% (v/w) yeast extract, 3% (v/v) glycerol, and 4% (v/v) ethanol. All media used in the SGA screens were supplemented with 2% galactose to limit cell–cell adhesion and allow more consistent pinning of the yeast arrays. Standard genetic and molecular methods were performed as previously described (Moreno *et al.* 1991).

The array strains used for the SL screens consisted of 91 transcription factor deletion strains previously constructed in Vachon *et al.* (2013). The query strains were constructed by performing a *NatMX4* marker switch of the *KanMX6* cassette in the array strains. PCR constructs containing the *NatMX4* cassette flanked with sequence homology to the outer regions of the *KanMX6* cassette were transformed into the array strains by a lithium acetate protocol and plated on YES + clonNAT medium. Transformants were confirmed by colony PCR and then crossed to wild type to obtain the *h^+^* mating type. Double transcription factor deletion strains were selected on YES medium containing both G418 and clonNAT.

In the SDL screens, the miniarray strains consisted of 279 Bioneer haploid mutants with deletion of genes that primarily encode for posttranslational regulators, including kinases, phosphatases, ubiquitin ligases, SUMO transferases, and chromatin remodeling enzymes (Supplementary Table 2). The query strains were created by transforming *pREP1* vectors overexpressing a unique transcription factor ORF with the *nmt1* promoter (Vachon *et al.* 2013) into the JK366 strain, which has the same auxotrophic background as the array strains. Deletion strains overexpressing the transcription factor ORF after mating were selected on PMG medium with 225 mg/L each of A, L, and U, as well as 300 mg/L of G418. The G418 concentration was increased to 300 mg/L in PMG medium to better select against strains that did not contain the *KanMX4* cassette.

To create an endogenously tagged *scr1-GFP* strain, the *EGFP* ORF and *KanMX4* cassette were PCR-amplified from the pYM27 plasmid (Janke *et al.* 2004). Regions homologous to either side of the 3′ end of the *scr1^+^* ORF were attached to the GFP sequence by PCR stitching. This construct was then inserted in-frame at the 3′ end of the *scr1^+^* ORF by lithium acetate transformation.

The *sds23* overexpression strain was constructed by inserting the *sds23^+^* ORF into the pREP1 vector by the In-Fusion cloning protocol (Takara Bio USA Inc.), followed by lithium acetate transformation into a *leu1-32* strain.

### SL screens

The SL screens involved crossing 38 transcription factor deletion query strains (*NatMX4*) with a transcription factor deletion array of 91 strains (*KanMX6*) and were based on the SpSGA methodology developed by Dixon *et al.* (2008). As a control, the *Δleu1::KanMX4* Bioneer strain was subjected to a marker switch with the *NatMX4* cassette and crossed to the transcription factor deletion array to obtain an estimate of the single mutant fitness of the array strains. SL screens were performed in 768 colony array format in which each transcription factor deletion array strain was represented 6 times with a border of *Δhis5* strains to control for spatial bias at the edges of the plate, as well as at least 1 blank spot. All media used in the SL SGA screens were supplemented with A, L, H, and U to ensure that growth was not affected in the control query strain. The query and array strains were mated by mixing on SPAS plates with a BM3 BioMatrix Robot (S&P Robotics, Inc.). The plates were incubated at 25°C for 3 days to allow mating and sporulation and then transferred to 42°C for 3 days to select for spores and remove unmated vegetative cells. The spores were germinated by pinning on YES medium and the vegetative cells were grown for 3 days at 30°C. The cells were subsequently pinned on YES medium containing G418 and clonNAT to select for double transcription factor deletion strains. In this final step, the entire array was pinned in duplicate to a final density of 1,536 colonies per plate in which each double mutant strain was represented a total of 12 times.

The plates were photographed, and strain fitness was determined by colony size using a spImager-M system and integrated software (S&P Robotics, Inc.). Normalization of colony size to correct for spatial biases, resulting from variation in the media or local environment on the plate, was performed with SGAtools (Wagih *et al.* 2013). The average fitness score of each double mutant strain was determined from individual scores of the 12 replicate colonies per screen across 3 replicate screens, as well as scores generated from other screens containing the reciprocal cross. The normalized fitness of the double mutant strain was compared to the fitness of the single mutant strains that were obtained by crossing the transcription factor deletion array to the *Δleu1::NatMX4* control query strain. Transcription factor genes located within 200,000 base pairs of the deleted gene in the query strain were not included in the genetic-interaction dataset because of linkage effects. None of the transcription factor genes were located within 200,000 base pairs of the *leu1^+^* control gene. The final fitness score was based on a multiplicative model in which the single mutant fitness weights were multiplied to generate a predicted double mutant fitness (Wagih *et al.* 2013). The threshold score used to identify negative genetic interactions was −0.185, because that was 2 standard deviations from the mean value.

### Random spore analysis

Random spore analysis (RSA) was performed to validate negative genetic interactions from the SGA screens. Parental strains were mated on SPAS medium, incubated for 3 days at 25°C, and then suspended in 0.5% glusalase solution for 6 h at 30°C to remove vegetative cells. The spores were washed twice in sterile water to remove the glusalase, diluted 1,000-fold, and plated at a ratio of 1:2:2:4 on YES, YES + G418, YES + clonNAT, and YES + G418 + clonNAT media (Dixon *et al.* 2008). The plates were incubated at 30°C for 3–5 days, depending on the growth of the single mutants, and the relative density of colonies was compared. Double mutant combinations were scored as synthetic lethal when fewer than 10 colonies were observed on YES + G418 + clonNAT plates, whereas combinations were considered moderate negative genetic interactions when the colony density was sparse relative to the fitness of the single mutants. Mild negative genetic interactions were scored when the double mutant colony density was high but could still be recognized as lower than the single mutants by blind selection.

### SDL screens

**Fig. 1.**
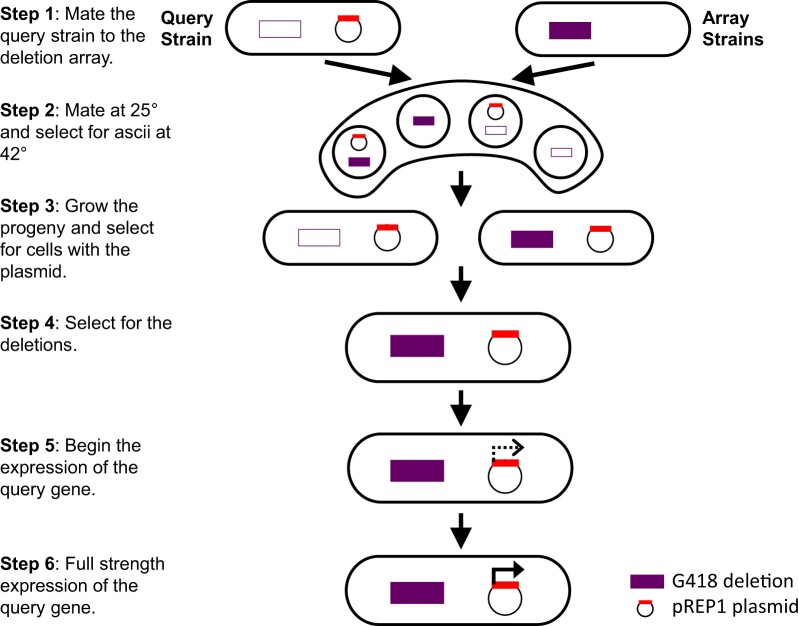
The SGA-based screening protocol for identifying SDL interactions in *S. pombe*. The 279 deletion array strains were arrayed on a single plate at a colony density of 384. The *nmt1*-driven overexpression query strain was crossed to the deletion miniarray in step 1. The selection of mated spores in step 2 was similar to the SGA protocol outlined by Dixon *et al.* (2008), with a 3-day incubation on SPAS plates at 25°C followed by another 3-day incubation at 42°C for mating and selection of spores, respectively. This was followed by a 5-day incubation in step 3 on EMM + AU medium supplemented with thiamin to allow for the spore germination and growth of vegetative cells. In step 4, the deletions were selected for on PMG + AU + G418 with thiamin before induction of the *nmt1* promoter. The selection of the deletion mutants and the induction of the *nmt1* promoter were performed in steps 5 and 6 to detect putative SDL interactions. PMG + AU + G418 was used to select for both the gene deletion and the plasmid while overexpressing the transcription factor target gene. The final colony size was imaged with the spImager-M system (S&P Robotics, Inc.) and scored using SGAtools (Wagih *et al.* 2013).

A systematic SDL screening method was developed in *S. pombe* by modifying the SGA procedure from Dixon *et al.* (2008) (Fig. 1). The SDL screens were conducted with a BM3 BioMatrix Robot (S&P Robotics, Inc.) and used to identify deletion backgrounds of regulator genes that result in lethality when combined with a transcription factor overexpression strain. The regulator gene deletions (miniarray strains) and the transcription factor overexpressor (query strain) were assembled in 384 colony array format on YES + G418 and on EMM + AU + thiamin plates, respectively, then incubated at 30°C for 3 days. The query strain was then crossed to the miniarray strains on SPAS medium to introduce the transcription factor overexpression vector into the regulator gene deletion strains. The SPAS plates were incubated for 3 days at 25°C for mating and then incubated at 42°C for 3 days to select for spores and kill unmated vegetative cells. The spores were subsequently transferred through pinning onto EMM + AU + thiamin medium and incubated for 5 days at 30°C to allow for germination. The colonies were then pinned onto PMG +AU + G418 + thiamin medium and incubated for 3 days at 30°C to select for the regulator gene deletion strains. This was followed by 2 rounds of pinning with a 3-day incubation on PMG + AU + G418 medium without thiamin at 30°C after each pinning to select for regulator gene deletion strains and allow induction of the *nmt1* promoter and overexpression of the transcription factor ORF. These 2 rounds of pinning reduced the carryover of cells from previous pinnings and allowed for better detection of SDL interactions (Supplementary Fig. 1). The final set of plates was then photographed, and colony sizes were determined using the spImager-M system (S&P Robotics, Inc.). As a control, an empty vector query strain was crossed to the miniarray strains to obtain an estimate of the single mutant fitness of the deletion strains. Normalized and final fitness scores were determined as described previously for the SL screens. For SDL interactions, a conservative cutoff value of −0.5 was selected to reduce false positives compared to a less stringent cutoff value of −0.3, which is normally considered a strong negative genetic interaction (Wagih *et al.* 2013).

### Serial dilutions

Serial spot dilutions were used to confirm putative SDL interactions. The transcription factor overexpression vector was retransformed into candidate regulator deletion strains, and the fitness was compared to the empty vector control, transcription factor overexpression strain, and the regulator deletion strain containing the empty vector. The comparisons were performed on EMM + AU medium in the presence and absence of thiamin after 3–5 days of growth at 30°C.

### Fluorescence microscopy

All fluorescence cell images in this study were captured with a Zeiss Imager Z1 microscope and AxioCam MRM digital camera (Zeiss, Thornwood, NY, USA). To examine potential additive cell cycle phenotypes from our SL screens, single and double transcription factor deletion strains were exponentially grown in YES medium at 30°C. The cells were then methanol-fixed and treated with DAPI (1 μg/mL) to stain the nuclei and calcofluor white (50 μg/mL) to stain the cell wall material. To identify potential regulators of transcription factors from our SDL screens, the intracellular localization and intensity of Scr1-GFP under the native promoter were compared in wild-type, *Δubr1*, *Δmub1*, *Δsds23*, and *Δamk2* cells. Strains were grown in logarithmic phase for 6 h in YES, YES low-glucose medium, EMM, and FIM, and then live cell images were acquired. The quantification of GFP signal intensity was determined for the entire cell area using ImageJ (v1.48, NIH). The corrected total cellular fluorescence was calculated as described by McCloy *et al.* (2014) for 30 cells in each of the 3 biological replicates. Three different locations per image were selected for background correction. Statistical significance of corrected total cellular Scr1-GFP fluorescence between wild type and the *Δubr1* and *Δmub1* cells was determined by a 2-tailed *t*-test.

### Flocculation assay

Normal and constitutive flocculation of deletion and control strains were assayed by inoculating ∼10^7^ cells/mL into 20 mL FIM and liquid EMM, respectively, and grown for at least 72 h at 30°C in a shaking incubator. The induction of flocculation by overexpression of *sds23^+^* was determined by incubating a *nmt1* promoter-driven *sds23^+^* strain on an EMM + thiamin plate overnight at 30°C, and cells (∼10^7^ cells/mL) were then transferred into 20 mL of liquid EMM and grown for 24 h at 30°C in a shaking incubator. To test if overexpression of *scr1^+^* could repress flocculation, a *nmt1* promoter-driven *scr1^+^* strain was streaked on an EMM plate without thiamin and incubated for 24 h at 30°C to allow induction of gene expression. Cells (∼10^7^ cells/mL) were then transferred into 20 mL of liquid FIM and grown for at least 72 h at 30°C in a shaking incubator. After incubation, 5 mL of cell cultures were transferred to 60 mm × 15 mm Petri dishes and placed on an orbital shaker for 10 min to promote floc formation. Images of flocs were acquired using a spImager-M system (S&P Robotics, Inc.). Three replicates of the flocculation assay were performed for each strain.

## Results and discussion

### SGA screen design

**Fig. 2.**
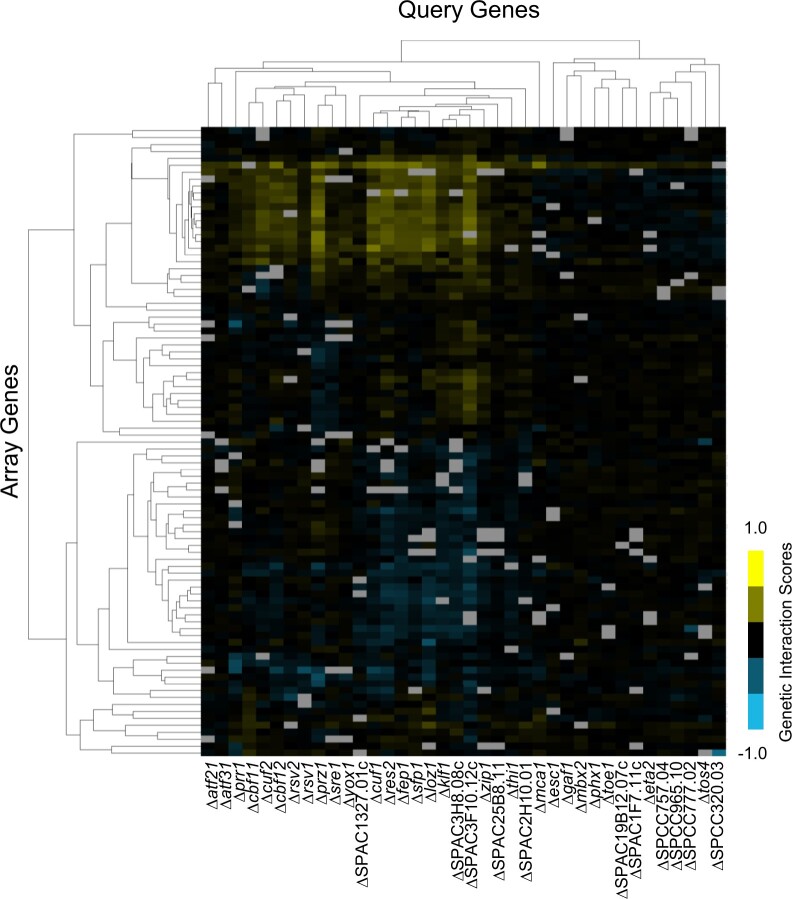
A heatmap of the genetic interactions between the 38 query and 91 array transcription factor deletion strains. The interaction scores are mapped to colors as indicated by the color bar at the bottom right, with negative scores in cyan are mostly clustered at the bottom and positive scores in yellow are mostly clutered together at the top right. The light gray squares indicate interactions that were omitted due to the possibility of gene linkage. All screens were performed with 3 biological replicates, and each array strain at 3 different locations on the plate per replicate.

An SL SGA screen was performed to identify genetic interactions between the sequence-specific transcription factor genes in *S. pombe*. Ninety-one nonessential transcription factor deletion mutants were used as the array strains, 38 of which were also selected as query strains (Supplementary Table 3). The query genes were involved in a range of biological processes, including the cell cycle, ion homeostasis, reproduction, stress response, and several with no annotated function. Of the 19 DNA-binding domain classes found among the sequence-specific transcription factors in *S. pombe*, 13 classes were represented in the 38 query strains (Supplementary Table 3). Thirty-eight query strains were crossed against the 91 array strains to produce 3,458 double mutant combinations (38 × 91) among the *S. pombe* transcription factor genes. This was reduced to 2,672 double-mutant combinations when we removed the double mutants obtained from reciprocal crosses, self-crosses, and those that were linked through genomic proximity (Fig. 2). This dramatically increased the number of double mutant combinations between transcription factor genes compared to those generated in previous whole genome SGA studies (Roguev *et al.* 2007; Ryan *et al.* 2012). The screens by Roguev *et al.* (2007) included crosses between 10 × 10 transcription factor genes resulting in 39 unique double mutant combinations, 34 of which overlapped with this study. The genome-wide screens by Ryan *et al.* (2012) included crosses between 27 × 37 transcription factor genes resulting in 834 unique double mutant combinations, 507 of which overlapped with this study. Our screens add a considerable number of novel double mutant combinations between transcription factor genes to those obtained in previous studies.

### Negative genetic interactions between *S. pombe* transcription factors

There were 46 negative genetic interactions observed in the 2,672 double mutant combinations generated in this study (1.72%) (Supplementary Table 4). The screens by Ryan *et al.* (2012) identified 29 negative genetic interactions in the 834 double mutant combinations of sequence-specific transcription factor genes (3.52%). The ability to compare hits between the 2 screens was limited by the fact that most of the negative genetic interactions observed were between double mutant combinations that were not created in the other study. Of our 46 negative genetic interactions, 3 were also identified as negative interactions by Ryan *et al.* (2012), 5 were not identified, and 38 were not comparable because the double mutant combinations were not created in their screen. Of the 29 negative genetic interactions identified by Ryan *et al.* (2012), the same 3 were also identified as negative interactions in this work, 18 were not identified, and 8 were not comparable because the double mutant combinations were not created in our screens. The 3 negative genetic interactions consistent between both studies were *tos4^−^* with *res2^−^*, *prz1^−^* with *sep1^−^*, and *yox1^−^* with *sep1^−^*.

The low overlap between the 2 screens may be due to differences inherent in experimental design, growth conditions, and analysis of the SGA/E-MAP methodologies. Our SGA screens employed heat shock to enrich for spores and select against vegetative cells, while the Ryan *et al.* (2012) E-MAP screens utilize cycloheximide resistance and 5-FOA to select against diploids and for *h^+^* haploids, respectively. The different stresses that cells are exposed to could have various effects on fitness for certain mutant strains, such as thermosensitive strains in our screens. Unlike the Ryan *et al.* (2012) screens, galactose was included in the media to reduce clumping of cells and obtain more consistent pinning in our screens. These differences, as well as methods to determine cutoff thresholds in fitness and interaction scores, could impact the overlap of the negative interactions. Indeed, a comparative study of large SGA and EMAP datasets in *S. cerevisiae* revealed low correspondence of fitness measurements and genetic-interaction scores between the 2 types of screens (Linden *et al.* 2011). These observations highlight the importance in validation of large-scale genetic-interaction screens by methods such as RSA or tetrad dissection to increase confidence in the data.

Among the 46 negative genetic interactions identified by SL SGA, 18 of the high confidence interactions were tested by RSA. These included 8 strongest interactions, 1 reciprocal interaction, 3 interactions identified in other large-scale SGA screens, as well as a subset of other interactions from the top half of the scores. RSA confirmed 12/18 negative genetic interactions identified in our SGA screens (Table 1 and Supplementary Fig. 2). The negative genetic interactions often had 1 single mutant that exhibited a greater growth defect. Of the 3 negative genetic interactions that overlapped with Ryan *et al.* (2012), 2 confirmed with RSA, while the interaction between *yox1^−^* and *sep1^−^* did not. The other negative genetic interactions that failed to confirm by RSA involved *scr1^−^*, where the *Δscr1* single mutant did not recover consistently from mating.

**Table 1.** Comparison of the interaction scores among the *S. pombe* transcription factors from the SGA screens, with the strength of the interactions observed by RSA for the 12 confirmed negative genetic interactions.

Query strain	Array strain	Interaction score	RSA score
*Δprr1*	*Δatf21*	−0.25	Lethal
*Δprr1*	*Δfil1*	−0.38	Lethal
*ΔSPCC320.03*	*ΔSPAC3C7.04*	−0.58	Moderate
*Δloz1*	*Δsre2*	−0.43	Moderate
*Δprz1*	*Δsep1*	−0.41	Moderate
*ΔSPAC3F10.12c*	*Δmug151*	−0.32	Moderate
*Δres2*	*Δace2*	−0.30	Moderate
*Δloz1*	*Δsep1*	−0.27	Moderate
*Δres2*	*Δtos4*	−0.25	Moderate
*ΔSPAC3F10.12c*	*ΔSPAC3H8.08c*	−0.22	Moderate
*Δprz1*	*ΔSPBC56F2.05c*	−0.34	Mild
*Δcbf12*	*Δace2*	−0.24	Mild

### Cell cycle phenotypes

Examination of the 12 negative interactions confirmed by RSA showed a prevalence of transcription factor genes with known roles in cell cycle regulation (Table 1 and Supplementary Fig. 2). These included regulators of cytokinesis (*ace2^+^* and *sep1^+^*) and S phase (*res2^+^* and *tos4^+^*) where their loss is known to cause defects in the progression of the cell cycle (Sipiczki *et al.* 1993; Miyamoto *et al.* 1994; Martin-Cuadrado *et al.* 2003; Kim, Tripathi, *et al.* 2020). Other genes such as *prr1*^+^, *prz1^+^*, and *cbf12^+^* do not exhibit a strong defect in the cell cycle when deleted but have direct links to known regulators of the cell cycle. For example, deletion of *prr1^+^* prevents G1 arrest under nitrogen deprivation, the gene product of *prz1^+^* is regulated by calcineurin that functions in cytokinesis and perturbation of *cbf12^+^* results in cytokinesis defects (Yoshida *et al.* 1994; Ohmiya *et al.* 2000; Hirayama *et al.* 2003; Prevorovský *et al.* 2009, 2015).

**Fig. 3.**
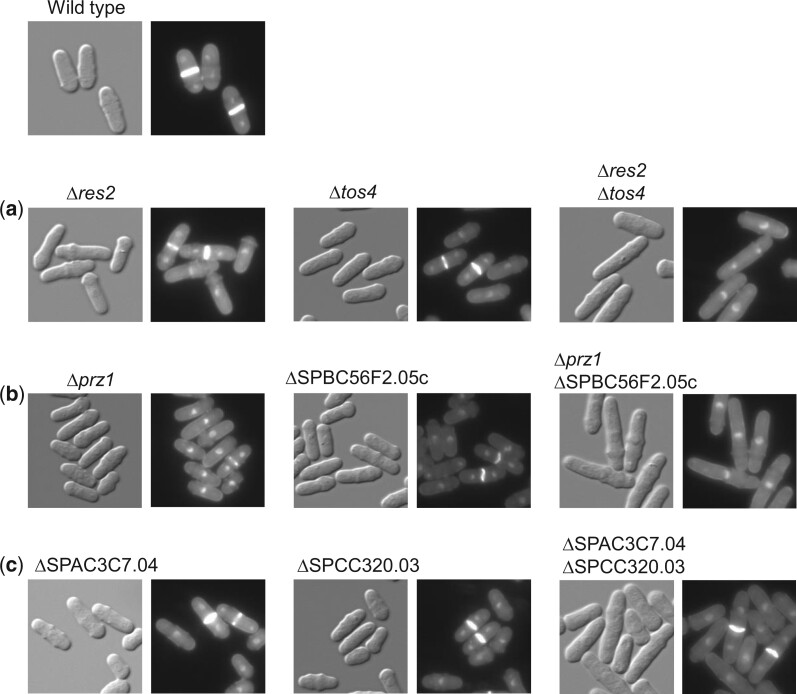
Transcription factor double mutants that result in a cell elongation phenotype. Cells were examined by differential interference contrast and fluorescence microscopy using DAPI and calcofluor white. a) The transcription factors Res2 and Tos4 have known roles in the cell cycle and the double deletion mutant cells are elongated more than either single deletion. b) The transcription factor Prz1 has been implicated in cell cycle regulation while SPBC56F2.05c is uncharacterized. The double mutant cells are elongated relative to the wild type and the single mutant controls. c) The transcription factors SPAC3C7.04 and SPCC320.03 are uncharacterized. Their function may be related to cell cycle regulation as the double mutant cells are elongated.

Three of the 10 double deletion mutants that displayed RSA-confirmed negative genetic interactions (the remaining 2 double mutants could not be examined due to SL interaction) had greater cell lengths than the single mutants, indicating a disruption of the cell cycle (Fig. 3). These additive phenotypes occurred in combinations of gene deletions of transcription factors, some of which have known roles in the cell cycle and others that remain uncharacterized (Fig. 3). For example, transcription factors Res2 and Tos4 both have known roles in the cell cycle, and their gene deletions shared a negative genetic interaction that resulted in elongated double mutant cells (Fig. 3a). Res2 is a component of the MBF transcription factor complex, which is responsible for the initiation of S phase, as well as proper DNA replication and damage checkpoint function (Miyamoto *et al.* 1994; Zhu *et al.* 1997; Dutta *et al.* 2008; Ivanova *et al.* 2013). Tos4 accumulates in the nucleus during S phase, and *tos4^+^* expression is regulated by the MBF complex (Kim, Tripathi, *et al.* 2020). The shared and related roles of Tos4 and Res2 in the S phase of the cell cycle could explain the negative genetic interaction between the 2 genes.

The negative genetic interaction from the deletion of SPBC56F2.05c and *prz1^+^* also resulted in double mutant cells that were more elongated than either single mutant (Fig. 3b). Prz1 is a calcineurin-responsive transcription factor that regulates multiple processes, which increases the difficulty in characterizing genetic interactions with Prz1, as they could be the result of its role in the cell wall, ion homeostasis, or reproduction (Chatfield-Reed *et al.* 2016). The observed negative genetic interaction from the deletion of *prz1^+^* and SPBC56F2.05c could be related to their potential roles in cytokinesis. Prz1 accumulates in the nucleus just prior to cell division, and SPBC56F2.05c shows a septation defect when overexpressed (Hirayama *et al.* 2003; Vachon *et al.* 2013).

The negative genetic interaction from the deletion of SPAC3C7.04 and SPCC320.03 also produced elongated double mutant cells (Fig. 3c). Neither SPAC3C7.04 nor SPCC320.03 have been well characterized, although SPAC3C7.04 has been shown to have elongated telomeres (Liu *et al.* 2009). The double mutant phenotype indicates a cell cycle delay when both genes are deleted, although the nature of their involvement in the cell cycle defect is not currently known.

The RSA-confirmed negative genetic interaction of the *Δloz1 Δsre2* double mutant (Supplementary Fig. 2) is not related to the cell cycle, given the primary function of these transcription factors in zinc and possibly lipid homeostasis, respectively (Miyamoto *et al.* 1994; Hughes *et al.* 2005; Dutta *et al.* 2008; Corkins *et al.* 2013). However, for other negative genetic interactions where only one of the transcription factors has a known cell cycle role, such as in *Δprr1 Δfil1* or *Δloz1 Δsep1* mutants (Supplementary Fig. 2), the deletion of the noncell cycle transcription factor could alter the physiology of the cell that results in perturbing the cell cycle. Supportive evidence of this hypothesis is the observation that perturbation of several fungal-specific Zn(2)-C6 transcription factor genes that often function in metabolism can result in cell elongation and cell cycle defects (Vachon *et al.* 2013).

### Conserved genetic interactions between yeast species

One aim of mapping the genetic interactions in yeast is to establish a conserved interaction network across species. The transcription factor genetic-interaction network has been partially explored in *S. cerevisiae* using SGA (Zheng *et al.* 2010). Thirty-eight of the *S. cerevisiae* sequence-specific transcription factor genes had 1 or more orthologs present in the *S. pombe* transcription factor array strains (Zheng *et al.* 2010; Wood *et al.* 2012). When accounting for multiple orthologs among those 38 genes, the possible overlap increased to 51 transcription factors, and a total of 1,228 double mutant combinations were scored in both screens. Using the cutoff selected by Zheng *et al.* (2010), there were 18 negative genetic interactions among the *S. cerevisiae* transcription factors that have *S. pombe* orthologs. This increased to 28 possible overlapping negative genetic interactions when accounting for those with multiple orthologs. These genetic interactions overlapped poorly between the 2 screens with only 1 conserved interaction. The *S. cerevisiae* transcription factor genes *MIG1* and *MIG2* shared a negative genetic interaction when deleted, which is orthologous to the negative genetic interaction between *rsv1^−^* and *scr1^−^* in *S. pombe* detected from our screens but failed to confirm by RSA due to inconsistency in recovery of the *Δscr1* single mutant (Supplementary Fig. 2). While Mig1p and Mig2p transcription factors repress a common set of target genes in the presence of glucose, the glucose repressive transcriptional roles of Rsv1 and Scr1 are not as redundant (Westholm *et al.* 2008). Rsv1 represses genes required for long-term survival under glucose starvation while Scr1 represses genes under low-glucose conditions (Saitoh *et al.* 2015; Kim, Cho, *et al.* 2020). The lower functional redundancy of *rsv1^+^* and *scr1^+^* may not confer a negative genetic interaction when both genes are deleted and suggests the occurrence of significant evolutionary changes in the function and target genes of these related transcription factors between the 2 yeasts. The limited overlap suggests that the sequence-specific transcription factor genetic-interaction networks between the 2 highly divergent yeast species have been substantially rewired. This is consistent with previous reports, which show significant divergence in *cis*-regulatory elements and gene expression within budding yeast species and with *S. pombe* (Sarda and Hannenhalli 2015; Chatfield-Reed *et al.* 2016; Tebung *et al.* 2016; Metzger *et al.* 2017).

### Transcription factor SDL screens

In *S. pombe*, overexpression of approximately two-thirds of transcription factor genes under the *nmt1* promoter causes reduced fitness (Vachon *et al.* 2013). This growth inhibition is likely a result of cellular toxicity from aberrant gene expression and physiological states when certain transcription factors become hyperactive when overexpressed. This is supported by systematic overexpression studies in *S. pombe* and *S. cerevisiae* that have identified direct target genes and binding specificity of several transcription factors by transcriptome profiling of overexpression strains that exhibit reduced fitness (Chua *et al.* 2006; Vachon *et al.* 2013). Based on these findings, deletion strains that further enhance the activity of transcription factors overproduced under the *nmt1* promoter are expected to exacerbate the cellular toxicity associated with transcription factor overexpression. These genetic interactions would manifest as SDL, which occur when overexpression of a gene results in an exacerbation of fitness in a certain deletion background compared to wild type. These SDL interactions would potentially represent genes that encode negative regulators of the transcription factor.

**Fig. 4.**
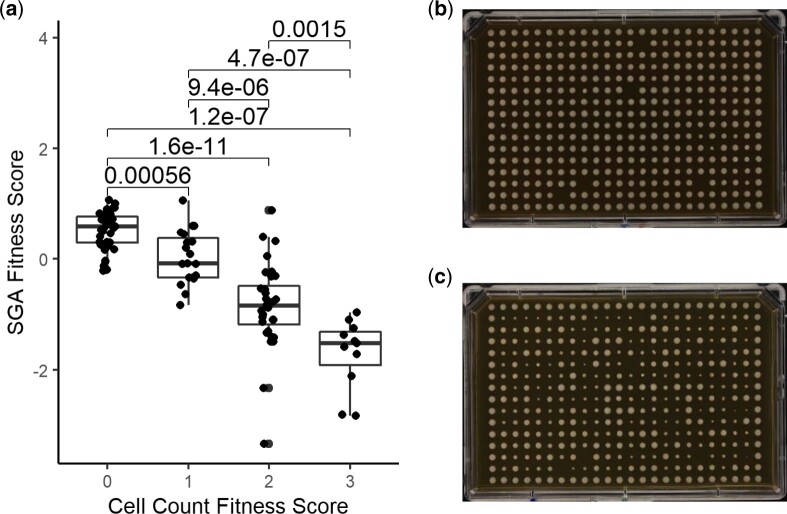
Correspondence of reduced fitness of transcription factor overexpression strains detected by robotic pinning and microscope visualization of cells/colony from Vachon *et al.* (2013). a) The relationship between the manual scores of cells/colony and the fitness scores of the transcription factor overexpression strains from SGA screening with significant differences observed between all 4 categories (*P*-values are from 2-tailed *t*-tests). The SGA fitness scores were based on 3 biological replicates. b) The colony sizes on a plate with thiamin after 3 days of growth. c) The colony sizes on a plate without thiamin after 3 days of growth (ectopic expression of the transcription factor gene).

We developed a systematic SDL screening method using SGA for *S. pombe* to identify potential regulators of transcription factors. The SDL screens involved query strains containing the *pREP1* vector with transcription factor ORFs overexpressed by the *nmt1* promoter. These were crossed with a miniarray containing 279 Bioneer haploid gene deletion strains of mainly posttranslational modifying enzymes (e.g. kinases, phosphatases, ubiquitin ligases, SUMO transferases, as well as chromatin remodeling factors) (Supplementary Table 2). Fourteen transcription factor overexpression strains were selected as queries for the SDL screens. These included 10 previously characterized transcription factors (Cbf11, Eta2, Sre2, Sfp1, Scr1, Toe1, Mbx1, Oxs1, Tos4, and Yox1), as well as 4 previously uncharacterized transcription factors (SPAC1F7.11c, SPAC19B12.07c, SPBC19G7.04, and SPBC530.08). The characterized transcription factors have been implicated in a variety of biological processes, including cell cycle regulation, glucose metabolism, pyrimidine salvage, diamide response, and flocculation (Kwon *et al.* 2012; Vachon *et al.* 2013; Saitoh *et al.* 2015; He *et al.* 2017; Kim, Tripathi, *et al.* 2020). Seven of the 14 transcription factors have predicted human orthologs (Cbf11, Eta2, Sre2, Sfp1, Mbx1, Oxs1, and SPAC19B12.07c). The transcription factor overexpression strains exhibited fitness defects ranging from mild to severe when compared to wild type. Robotic pinning of the transcription factor overexpression strains in the absence of thiamin exhibited fitness defects that agreed with those previously observed in Vachon *et al.* (2013), with the exception of Toe1, which showed a milder fitness defect when pinned (Fig. 4). Five additional transcription factors (Prz1, Map1, Fil1, Grt1, and Gaf1) were also selected as queries for the SDL screens but were omitted due to inconsistent growth on multiple replicates.

**Fig. 5.**
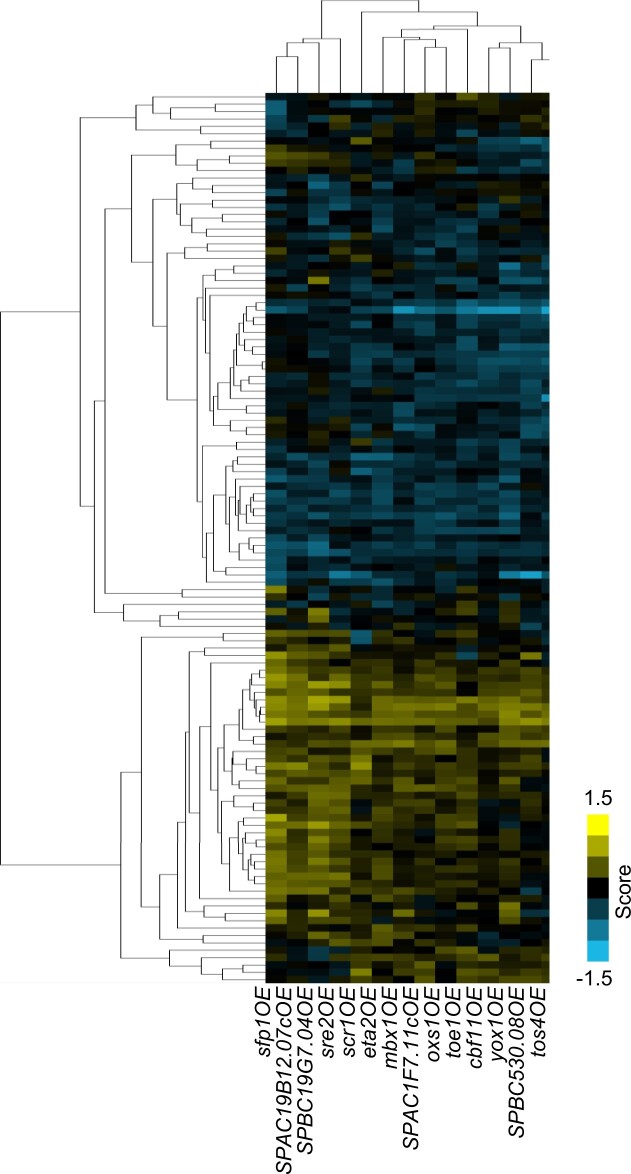
The *S. pombe* genetic interactions from SDL screens of transcription factor overexpression query strains and deletion array strains of posttranslational modifying enzymes. The 121 of 279 genes when deleted that showed a genetic interaction score of either >0.5 or <−0.5 with one of the 14 transcription factor overexpression query strains are shown in the heat map. Each column represents an overexpression query strain and each row represents a deletion array strain. Positive genetic interactions are mostly clustered in the bottom part of the figure and indicated in yellow and negative genetic interactions (SDL) are clustered at the top of the figure and indicated in cyan. The screens were performed with 3 biological replicates.

We detected 195 putative SDL interactions from 3,906 double-mutant combinations from our SDL SGA screens (4.99%) (Fig. 5 and Supplementary Table 6). Fifty-one of the 195 SDL interactions were selected for confirmation by serial dilution, predominantly from the top half of the interaction scores or those involved with transcription factors of specific interest to our lab. Of the SDL interactions tested, 58.8% were confirmed by serial dilution and 23.5% were identified as false positives, which compares favorably to previous screens (Liu *et al.* 2009; Sharifpoor *et al.* 2012; Youn *et al.* 2017) (Supplementary Table 6). The remaining SDL interactions could not be confirmed due to either severe fitness defects in the single deletion mutants or because the double mutant combinations exhibited severe fitness defects when grown on the thiamin-containing control plates. The SDL combinations that exhibited a fitness defect in the control condition may be the result of leaky expression of the *nmt1* promoter in the presence of thiamin and a strong genetic interaction with the deletion mutant, which does not tolerate any degree of transcription factor overexpression.

There were 7 previously characterized interaction pairs generated in the screens and 4 that we captured as SDL interactions: *scr1^+^* overexpression with Δ*sds23*, as well as *yox1^+^* overexpression with Δ*cds1*, Δ*cdt2*, and Δ*gad8* (Gómez-Escoda *et al.* 2011; Stewart *et al.* 2011; Matsuzawa *et al.* 2012; Saitoh *et al.* 2015; Cohen *et al.* 2016; Gaspa *et al.* 2016). Both Sds23 and Cds1 are kinases that directly negatively regulate their respective transcription factors. The Cds1 kinase phosphorylates Yox1 and inhibits its interaction with the MBF complex in response to DNA damage (Gómez-Escoda *et al.* 2011), while the Sds23 kinase phosphorylates Scr1 to prevent its nuclear translocation under low-glucose conditions (Saitoh *et al.* 2015). No interaction was detected between *scr1^+^* overexpression and the loss of another previously confirmed upstream regulator of Scr1, *ssp2^+^*, despite it having a similar regulatory role on Scr1 nuclear localization as observed in the *Δsds23* strain (Matsuzawa *et al.* 2012). The deletion of *cdt2^+^* or *gad8^+^* has been shown to have a role in the regulation of the MBF complex and Yox1 through indirect and less well-characterized methods (Cohen *et al.* 2016; Gaspa *et al.* 2016). However, the Cul4-RING E3 complex subunit Ddb1, from the same complex as Cdt2, did not have a synthetic lethal interaction with *yox1^+^* overexpression when the gene was deleted (Gaspa *et al.* 2016). Finally, the activation of the SREBP transcription factor Sre2 by the ubiquitin ligase Dsc1 was not captured by the screen because it is not a SDL interaction and, therefore, not expected to be detected in this screen (Stewart *et al.* 2011).

### Novel SDL interactions of *scr1^+^*

**Fig. 6.**
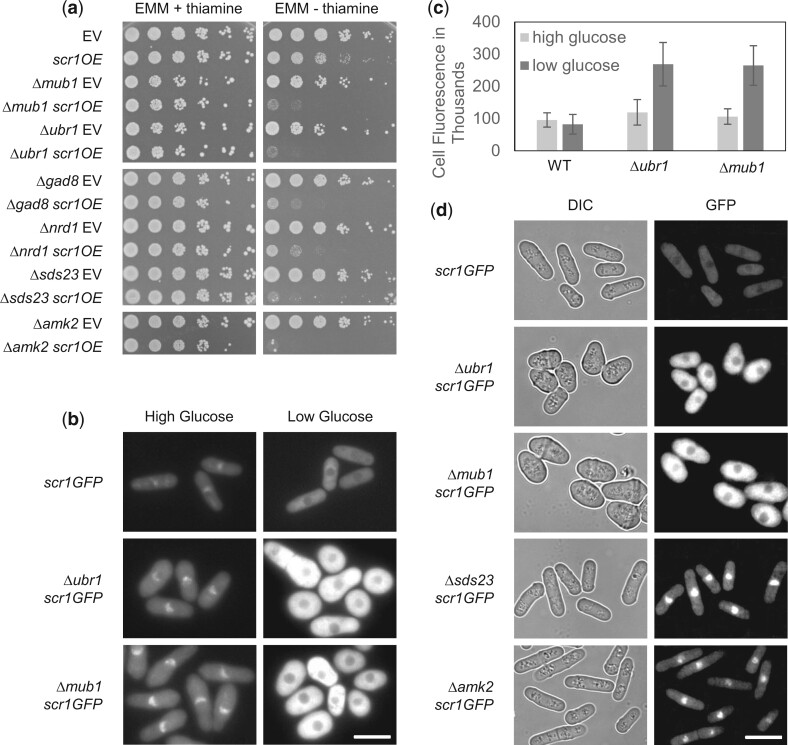
SDL interactions of *scr1^+^*. a) Confirmation of SDL interactions with *scr1^+^* by serial dilution. b) Fluorescence microscopy images of Scr1-GFP under either high- or low-glucose conditions in wild-type, *Δubr1*, and Δ*mub1* strains. c) The quantification of the Scr1-GFP total corrected cellular fluorescence in the 3 corresponding strains at the 2 different concentrations of glucose. The Scr1-GFP level in low glucose was significantly higher in the *Δubr1* and Δ*mub1* strains than in wild type (*P* < 0.0001). The Scr1-GFP level in high glucose was significantly higher in the *Δubr1* strain than in wild type (*P* = 0.0072). The total corrected cellular fluorescence values were calculated as described (McCloy *et al.* 2014) and represent 30 cells over 3 biological replicates. The error bars represent the standard error of the mean. d) Fluorescence and light microscopy of cells expressing Scr1-GFP under control of its native promoter in FIM for 6 h. Scr1-GFP is excluded from the nucleus in wild-type cells but shows nuclear localization in Δ*sds23* or Δ*amk2* cells. Scr1-GFP is upregulated in *Δubr1* or Δ*mub1* cells. All fluorescent cell images were acquired using the same exposure time. Bar = 10 µm.

The Scr1 transcription factor functions in catabolite repression by inhibiting transcription of target genes such as *inv1^+^*, *fbp1^+^*, *gld1^+^*, and *ght5^+^* in the presence of high glucose (Tanaka *et al.* 1998; Janoo *et al.* 2001; Matsuzawa *et al.* 2010; Saitoh *et al.* 2015). Scr1 activity is regulated in part by its intracellular localization, where it is predominantly nuclear when wild-type cells are grown in rich medium but remains mainly cytoplasmic under low-glucose conditions (Saitoh *et al.* 2015). In addition to *sds23^−^*, we discovered 5 other genes that exhibited confirmed SDL interactions with *scr1^+^* overexpression when deleted (Fig. 6 and Supplementary Table 6). Two of these genes, *amk2^+^* and *gad8^+^*, encode kinases that are known to be responsive to glucose levels (Valbuena and Moreno 2012; Hatano *et al.* 2015). Gad8 has been shown to have a role in the proper localization of Ght5, which encodes a hexose transporter in the plasma membrane, and whose gene precursor (*ght5^+^*) is regulated by Scr1 (Matsuzawa *et al.* 2012; Saitoh *et al.* 2015). Two other gene deletions that shared a SDL interaction with overexpression of *scr1^+^* were Δ*ubr1* and Δ*mub*1. Ubr1, a putative E3 ubiquitin ligase, appears orthologous to *S. cerevisiae* protein Ubr2. Ubr2 interacts in a protein complex containing Rad6 and Mub1, orthologous to *S. pombe* Mub1, to degrade its protein targets Rpn4, Sml1, and Dsn1 (Ju *et al.* 2008; Andreson *et al.* 2010; Akiyoshi *et al.* 2013). We next determined whether these SDL genes could regulate the intracellular localization and abundance of Scr1 by examining natively expressed Scr1-GFP in the corresponding deletion strains in both high (3%) and low (0.08%) glucose media. Interestingly, both *Δubr1* and *Δmub1* backgrounds displayed a significantly higher amount of Scr1-GFP relative to wild type under low-glucose conditions (*P* < 0.0001) (Fig. 6, b and c). While differences in high-glucose conditions were not as pronounced, Scr1-GFP levels in the Δ*ubr1* cells were still significantly higher than wild type (*P* = 0.0072) (Fig. 6, b and c). Altogether, these data suggest that Scr1 may be degraded in response to its inactivation in low glucose by Ubr1 and Mub1.

**Fig. 7.**
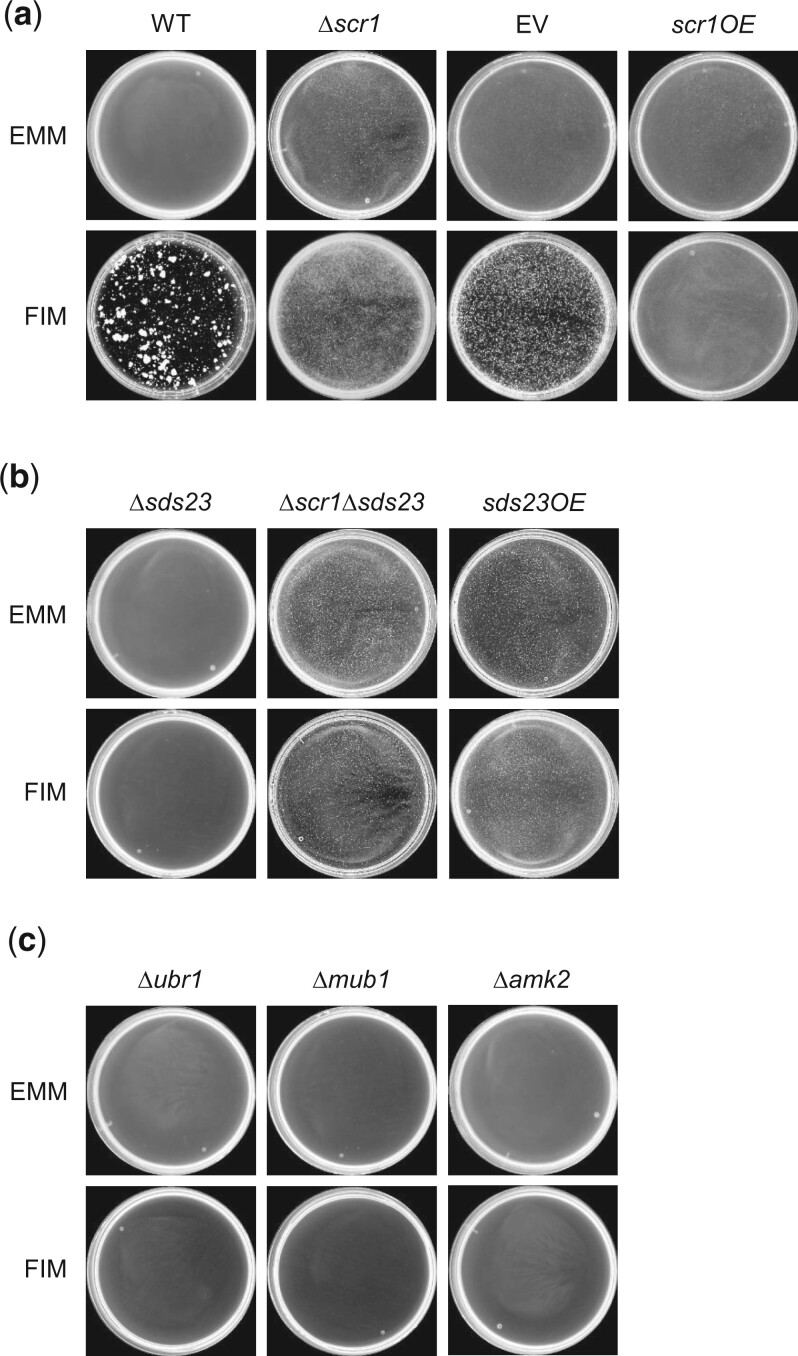
Flocculation assays of *scr1^+^* and SDL strains. a) Scr1 is a repressor of flocculation. The Δ*scr1* strain shows constitutive flocculation in liquid EMM while overexpression of *scr1^+^* with the *nmt1* promoter prevents flocculation in FIM. b) Sds23 is an activator of flocculation. The Δ*sds23* strain fails to flocculate in FIM while overexpression of *sds23^+^* with the *nmt1* promoter induces flocculation in liquid EMM. The nonflocculent phenotype of the Δ*sds23* strain is unable to suppress the constitutive flocculation of Δ*scr1* cells. c) The SDL strains *Δubr1*, Δ*mub1*, and Δ*amk2* fail to flocculate in FIM. Strains were grown in liquid EMM and FIM at 30°C for 2 and 5 days, respectively. Five milliliters of cell cultures were transferred to Petri dishes, placed on an orbital shaker for 10 min to promote floc formation and imaged with a spImager-M system (S&P Robotics, Inc.).

During this study, we observed that the Δ*scr1* strain formed small flocs in liquid EMM, indicating that Scr1 functions in the repression of flocculation under noninducing conditions (Fig. 7a). This novel function of Scr1 was further supported by the ability of *scr1^+^* overexpression to prevent flocculation of wild-type cells in FIM (Fig. 7a). Based on this discovery, we next proceeded to utilize the flocculent phenotype of the Δ*scr1* strain to validate and investigate the nature of the *scr1^+^* SDL interactions. The PP2A inhibitor Sds23 has been shown to have a role in cell proliferation under low-glucose conditions by regulating the nuclear localization of Scr1 (Saitoh *et al.* 2015). Loss of *sds23^+^* results in poor growth and nuclear localization of Scr1 under low-glucose conditions (Saitoh *et al.* 2015). These findings suggest that Sds23 inhibits Scr1 function by nuclear exclusion and accessibility to the promoter of its catabolite repression target genes during glucose deprivation. If Sds23 antagonizes the function of Scr1, then the former would function as a positive regulator of flocculation. Consistent with this hypothesis is the observation that *sds23^+^* overexpression is sufficient to induce flocculation in liquid EMM while loss of *sds23^+^* prevents flocculation in FIM (Fig. 7b). Examination of Δ*scr1*Δ*sds23* cells revealed the occurrence of flocculation in liquid EMM, which is also supportive of the role of Sds23 in inhibiting Scr1 function (Fig. 7b). We also examined the intracellular localization of Scr1 in cells induced to flocculate in FIM. Like glucose deprivation, Scr1 showed nuclear exclusion in wild-type cells grown in FIM but was predominantly nuclear in the Δ*sds23* background (Fig. 6d). Altogether, these results indicate that Scr1 represses flocculation under noninducing conditions, while Sds23 is involved in inhibiting Scr1 activity by nuclear exclusion during flocculation. Efforts are underway to identify the direct target genes of Scr1 that function in the repression of flocculation.

We hypothesized that hits recovered from our SDL screens of transcription factor genes are likely to represent negative regulators. If this was the case, then the SDL hits of *scr1^+^* may not flocculate in FIM as observed when *sds23^+^* was deleted. We found that the loss of *ubr1^+^*, *mub1^+^*, or *amk2^+^* prevented flocculation in FIM (Fig. 7c). Like *sds23^+^*, loss of *amk2^+^* resulted in nuclear localization of Scr1 in FIM, while intracellular expression levels of Scr1 were increased in a *ubr1* or *mub1* deletion background (Fig. 6d). The changes in nuclear localization in the Δ*amk2* background are consistent with the known binding of Amk2 with Ssp2, as the loss of *ssp2^+^* has previously been shown to result in aberrant nuclear expression of Scr1 in low-glucose conditions (Matsuzawa *et al.* 2012; Rallis *et al.* 2017). In contrast, Δ*gad8* and *Δnrd1* strains exhibited flocculation in FIM (data not shown). Therefore, 4 of the 6 confirmed SDL interactions of *scr1^+^* appear to function as putative negative regulators of Scr1, either through degradation by ubiquitination or nuclear exclusion.

In both *S. pombe* and *S. cerevisiae*, there is evidence that phosphorylation is a necessary precursor to ubiquitination by the Ubr1/Ubr2 complex. In *S. cerevisiae*, Sml1, an inhibitor of ribonucleotide reductase, is degraded by the Rad6-Ubr2-Mub1 ubiquitin ligase complex when it is phosphorylated upon exposure to DNA damage (Andreson *et al.* 2010). In *S. pombe*, Ubr1 is involved in the degradation of the transcription factors Mei2 and Pap1 (Kitamura *et al.* 2001, 2011; Marte *et al.* 2020). Mei2 degradation is dependent on phosphorylation by Pat1 and is performed by Ubr1 in conjunction with the E2 ubiquitin-conjugating enzyme Rhp6, the ortholog of the E2 enzyme present in the *S. cerevisiae* complex (Kitamura *et al.* 2001). A similar mechanism may occur in the degradation of Scr1 as it is phosphorylated in response to low-glucose levels in the cell (Matsuzawa *et al.* 2012).

### Novel SDL interactions of cell cycle transcription factor genes

There was functional enrichment of biological processes among the genes that had a SDL interaction with *tos4^+^* overexpression using the GO::TermFinder (Boyle *et al.* 2004). These SDL genes were involved in ubiquitination of histone H2B (GO: 0033523: corrected *P*-value = 0.00228). Tos4 is involved in the DNA damage response and its expression is tightly coupled with S phase (Aligianni *et al.* 2009; Bastos de Oliveira *et al.* 2012; Kim, Tripathi, *et al.* 2020). The histone modification genes that interacted with the *tos4^+^* overexpression strain include all 4 components of the histone H2B ubiquitin ligase complex (HULC): Brl1, Rhp6, Brl2, and Shf1. The HULC is responsible for the mono-ubiquitination of H2B lysine 119 and is associated with heterochromatin, enhanced silencing of several genes, and increased pol II occupancy at the centromeric repeats (Tanny *et al.* 2007; Zofall and Grewal 2007). Even more interesting was an association between the *S. cerevisiae* HULC subunit Bre1, an ortholog of *S. pombe* Brl1, and the origin of DNA replication (Trujillo and Osley 2012). Trujillo and Osley (2012) concluded that a loss of Bre1 caused a lack of H2B ubiquitination at the origin of replication, which resulted in slower S-phase progression due to a failure to assemble or stabilize the nucleosomes. Deletion of the HULC genes in combination with *tos4^+^* overexpression could be further disrupting the normal progression of S phase.

Yox1 functions as a repressor of the MBF transcription factor complex, which regulates target genes important in the G1/S transition of the cell cycle (Aligianni *et al.* 2009). There were 10 genes that, when deleted, shared a SDL interaction with *yox1^+^* overexpression that we confirmed by serial dilution (Supplementary Table 6). Eight of the 10 SDL interactions involved genes annotated with a function or abnormal phenotype related to the mitotic cell cycle, although this enrichment was not statistically significant (Tanaka and Okayama 2000; Tallada *et al.* 2002; Goshima *et al.* 2003; Hayles *et al.* 2013; Graml *et al.* 2014). These included genes encoding the kinases Oca1, Pef1, and Cdr1, with the latter 2 involved in the regulation of the G1/S and G2/M transition, respectively (Coleman *et al.* 1993; Tanaka and Okayama 2000). Despite confirmation of the SDL interactions by serial dilution, we did not detect additive effects in cell morphology when either *pef1^+^* or *cdr1^+^* was deleted in combination with *yox1^+^* overexpression (data not shown). Interestingly, the *Δpef1* and *Δcdr1* strains also share a synthetic negative genetic interaction with the *Δyox1* strain (Ryan *et al.* 2012). Altogether, these results suggest that the presence of either gene is crucial when *yox1^+^* is aberrantly expressed.

## Conclusions

In this study, we demonstrate that SGA-based SL and SDL screens of *S. pombe* transcription factor genes are effective in identifying new biological roles and regulators not easily revealed by analyses of single deletion and overexpression strains. Our SL screens more than double the current number of transcription factor double mutants analyzed, and 46 negative genetic interactions were recovered. Our screens partially overlapped with previous studies, perhaps due to the differences in methodology between the SpSGA method and pombe epistasis mapper system (Roguev *et al.* 2007; Dixon *et al.* 2008; Roguev *et al.* 2018). Several of these negative genetic interactions displayed a cell elongation phenotype involving uncharacterized transcription factors indicating potential novel roles in cell cycle regulation.

We also developed a modified SGA method for high-throughput screening of SDL interactions in *S. pombe* and demonstrated its utility in identifying upstream regulators of transcription factors. The SDL screens were able to identify known regulators of transcription factors, including the Sds23 and Cds1 kinases upstream of the Scr1 and Yox1 transcription factors, respectively, as well as Ubr1 and Mub1 as novel putative regulators of Scr1. These regulators appear to be repressors of their associated transcription factors, indicating that these SDL interactions may be due to an increase in aberrant regulation of target genes compared to the transcription factor overexpression strain alone. The SDL screens had limitations that were likely the result of the leakiness of the *nmt1* promoter, causing several transcription factor overexpression strains (*prz1^+^*, *map1^+^*, *fil1^+^*, *grt1^+^*, and *gaf1^+^*) to be omitted from the final analysis. Defects in growth, cell adhesion, or mating efficiency could alter the effectiveness of the robotic pinning or mating steps of the SGA procedure. In the future these complications could be alleviated by using a weaker promoter such as *nmt41* or *nmt81* to overexpress the transcription factor gene or by integrating the pREP plasmid into the genome (Youn *et al.* 2017). This would reduce the toxicity of the gene overexpression alone and increase the number of cell lines available for use as query strains and further increase the utility and scope of the screen.

## Data availability

Strains and plasmids are available upon request. The authors affirm that all data necessary for confirming the conclusions of the article are present within the article, figures, tables, and supplementary materials.

Supplemental material is available at *G3* online.

## Funding

This work was supported by grants from the Natural Sciences and Engineering Research Council of Canada (RGPIN-2016-06496) and Canada Foundation for Innovation (16855).

## Conflicts of interest

None declared.

## Supplementary Material

jkac194_Figure_S1

jkac194_Figure_S2

jkac194_Table_S1

jkac194_Table_S2

jkac194_Table_S3

jkac194_Table_S4

jkac194_Table_S5

jkac194_Table_S6
