# Supplementary material for: Genetic-interaction screens uncover novel biological roles and regulators of transcription factors in fission yeast
Source: G3 (Bethesda). 2022 Aug 4;12(9):jkac194. doi: 10.1093/g3journal/jkac194 (PMC9434175; doi:10.1093/g3journal/jkac194)
Supplement: jkac194_Figure_S1 [file jkac194_figure_s1.pdf]

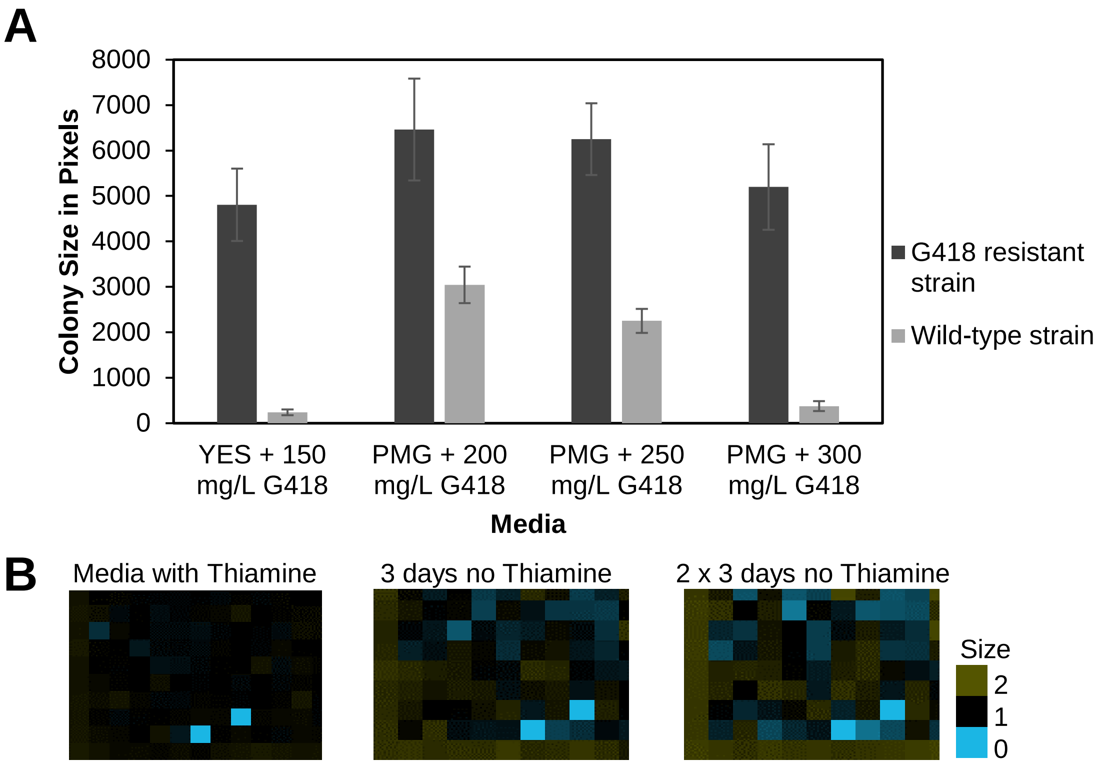

Figure S1: Experiments that determined the variables used for selection of the deletion mutants and induction of the overexpression plasmid. Minimal medium is required to maintain leucine selection of the transcription factor overexpression plasmid during the *S. pombe* SDL procedure. Standard minimal medium is not conducive to G418 selection, so Pombe Minimal Glutamate (PMG) medium was used instead. A) Several trials were performed to test the impact of the minimal media on G418 selection. PMG medium reduces the G418 sensitivity of the strains without the kanamycin-resistance cassette, relative to the fitness observed in rich medium. Increasing the concentration of G418 counteracts the increased growth and improves the selection. Each drug concentration was performed with 48 technical replicates on a plate and the error bars show standard error. B) The *nmt1* promoter is induced by the absence of thiamine in the media. The colony size is reduced after three days on plates without thiamine as seen in the difference between the first two figures. Three additional days on fresh minus thiamine plates increases the growth defect even further. The thiamine colony size measurements were performed with two biological replicates.
