# Supplementary material for: Genetic-interaction screens uncover novel biological roles and regulators of transcription factors in fission yeast
Source: G3 (Bethesda). 2022 Aug 4;12(9):jkac194. doi: 10.1093/g3journal/jkac194 (PMC9434175; doi:10.1093/g3journal/jkac194)
Supplement: jkac194_Figure_S2 [file jkac194_figure_s2.pdf]

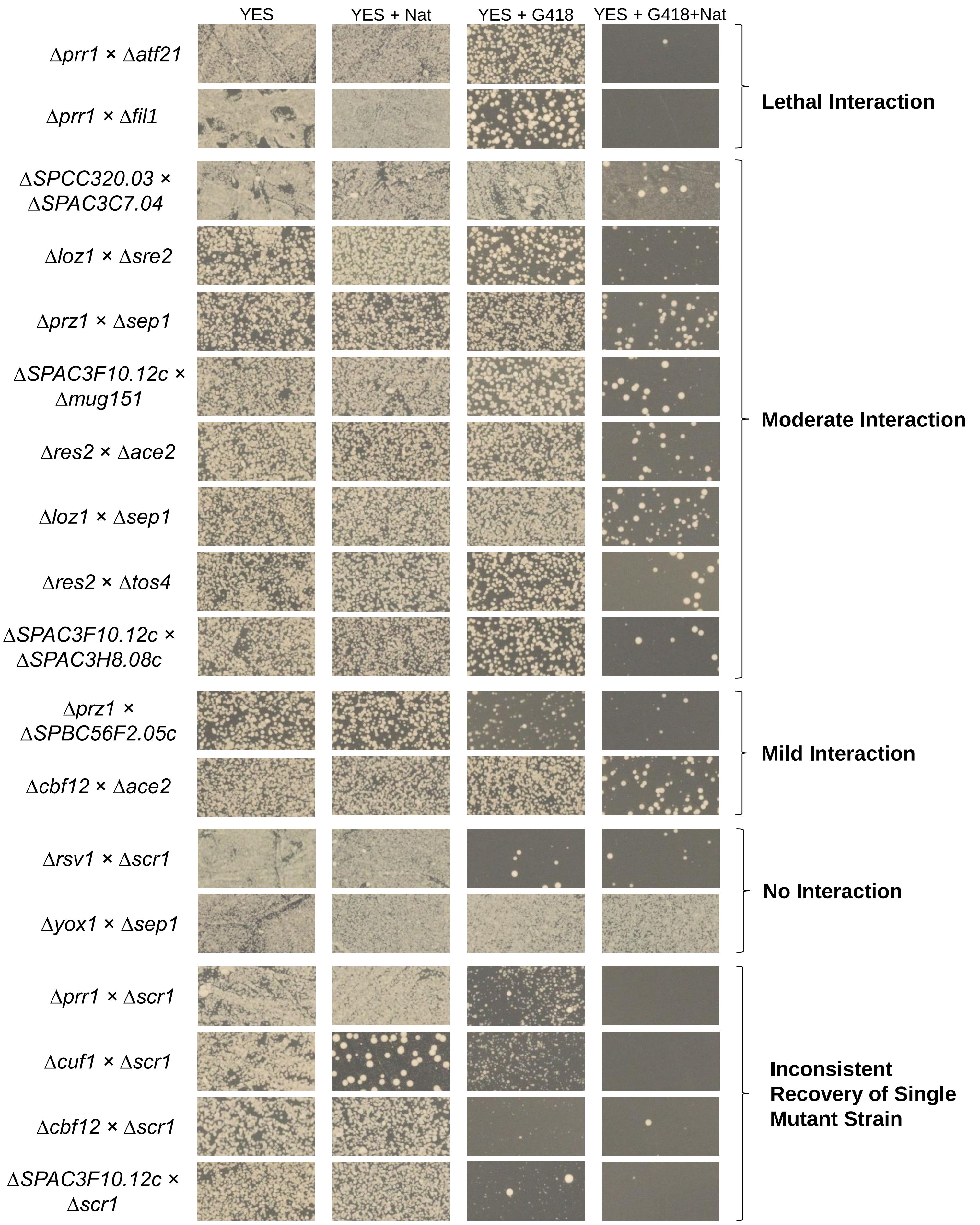
YES
YES + Nat
YES + G418
YES + G418+Nat
Δprr1 × Δatf21
Δprr1 × Δfil1
Lethal Interaction
ΔSPCC320.03 × ΔSPAC3C7.04
Δloz1 × Δsre2
Δprz1 × Δsep1
ΔSPAC3F10.12c × Δmug151
Δres2 × Δace2
Δloz1 × Δsep1
Δres2 × Δtos4
ΔSPAC3F10.12c × ΔSPAC3H8.08c
Moderate Interaction
Δprz1 × ΔSPBC56F2.05c
Δcbf12 × Δace2
Mild Interaction
Δrsv1 × Δscr1
Δyox1 × Δsep1
No Interaction
Δprr1 × Δscr1
Δcuf1 × Δscr1
Δcbf12 × Δscr1
ΔSPAC3F10.12c × Δscr1
Inconsistent Recovery of Single Mutant Strain

Figure S2: The genetic interactions confirmed by RSA. A) The lethal interaction. Interactions were considered lethal if there were fewer than 10 colonies on the YES+G418+Nat plate containing the double mutant. B) The moderate negative interactions. C) The mild negative interactions. Negative interactions were considered mild when the colony density on the YES+G418+Nat plate was high, but still easily chosen as the lowest density plate without prior knowledge of which plates contained which drugs. D) Interactions that did not confirm by RSA. E) Interactions between Δ*scr1* and other strains did not confirm because the Δ*scr1* mutant did not consistently grow after mating.
